# Non-criteria antiphospholipid antibodies in antiphospholipid syndrome: Diagnostic value added

**DOI:** 10.3389/fimmu.2022.972012

**Published:** 2022-10-26

**Authors:** Xiangjun Liu, Lei Zhu, Hongjiang Liu, Qingmeng Cai, Zelin Yun, Feng Sun, Yuan Jia, Jianping Guo, Chun Li

**Affiliations:** ^1^ Department of Rheumatology and Immunology, Peking University People’s Hospital, Beijing, China; ^2^ Department of Clinical Laboratory, Affiliated Nantong Rehabilitation Hospital of Nantong University, Nantong, China

**Keywords:** antiphospholipid syndrome, non-criteria antiphospholipid antibodies, thrombosis, APhL, aPS/PT antibody

## Abstract

**Objective:**

Non-criteria antiphospholipid antibodies (aPLs) increase the diagnostic value for antiphospholipid syndrome (APS) and contribute to better recognition of seronegative APS (SNAPS). However, the clinical utility and the diagnostic value of non-criteria aPLs are inconsistent. This study aimed to investigate the prevalence and clinical significance of 7 non-criteria aPLs in a large APS cohort.

**Methods:**

Seven non-criteria aPLs, including anti-phosphatidylserine/prothrombin (aPS/PT) antibodies IgG/IgA/IgM, anti-phosphatidylethanolamine antibodies (aPE) IgG/IgA/IgM, anti-Annexin V antibodies (aAnnexinV) IgG/IgA/IgM, anti-phosphatidylserine antibodies (aPS) IgM, aPS IgG, antibodies directed against a mixture of phospholipids (APhL) IgG, and APhL IgM were tested among 175 patients with APS, 122 patients with other autoimmune diseases (as disease controls), and 50 healthy controls.

**Results:**

In the present study, the highest prevalence of non-criteria aPLs was seen in aAnnexinV (58.86%). APhL IgG and aPS IgM showed the highest specificity (95.35%) and aPS/PT showed the highest Youden index (0.3991) for the diagnostic value of APS. The aAnnexinV also showed the highest prevalence in SNAPS (43.3%), followed by APhL IgM (21.7%), aPE (16.7%) and aPS/PT (16.7%). APhL IgG, aPS/PT, and aPS IgG showed positive association with thrombotic events in APS patients [APhL IgG: odds ratio (OR) = 2.26, 95% confidence interval (CI) 1.18-4.34, *p* = 0.013; aPS/PT: OR = 2.48, 95% CI: 1.32-4.69, *p* = 0.004; aPS IgG: OR = 1.90, 95% CI 1.01-3.60, *p* = 0.046; respectively). The inclusion of the non-criteria aPLs increased the accuracy of APS diagnosis from 65.7% to 87.4%.

**Conclusion:**

Our data provide evidence that adding the non-criteria aPLs can improve the diagnostic accuracy in APS. APhL IgG, aPS/PT, and aPS IgG may be potential biomarkers to predict the risk of thrombosis in APS.

## Introduction

Antiphospholipid syndrome (APS) is a systemic autoimmune disorder characterized by arterial and venous thrombosis and/or pregnancy morbidity with the presence of persistent antiphospholipid antibodies (aPLs) (1). According to the 2006 Sydney Classification criteria for definite APS (2), the IgG/IgM anticardiolipin antibodies (aCL), anti-β2-glycoprotein I antibodies (aβ2GPI), and lupus anticoagulant (LA) were defined as criteria aPLs. The three criteria aPLs are not only critical components in APS classification, but are also considered risk factors for thrombosis or pregnancy morbidity in APS (3, 4). They are also associated with APS “non-criteria” manifestations (5). All three criteria aPLs were included in two widely accepted risk score systems, i.e., APL-S (6) and the Global APS score (7). However, some patients exhibit clinical manifestations highly suggestive for the diagnosis of APS but persistently negative for criteria aPLs. These patients are defined as seronegative APS (SNAPS) (8).

To date, several non-criteria aPLs have been investigated to identify SNAPS better. The autoantigens specificity of these non-criteria aPLs includes different phospholipids, phospholipid binding proteins, and coagulation factors (9, 10). There are more than 30 known non-criteria aPLs in APS (11, 12). Among these, anti-phosphatidylserine/prothrombin antibodies (aPS/PT), aβ2GPI Domain I, IgA of aβ2GPI and aCL were highly specific for the identification of APS patients and have been the subject of previous investigations (9, 13–17). Of the non-criteria aPLs, aPS/PT are also included in the GAPSS and APL-S for risk stratification in APS patients (6, 7). Thus, the aPS/PT and aβ2GPI Domain I have been regarded as “first-line” non-criteria aPLs (18). However, the clinical significance of other non-criteria aPLs have not yet been investigated. These aPLs are still controversial because most existing studies evaluated only one or just a few non-criteria aPLs using different diagnostic assays, and have different study designs.

To better understand clinical significance of the non-criteria aPLs in APS, we evaluated the diagnostic value of seven non-criteria aPLs and their association with APS subphenotypes in a large APS cohort.

## Methods

### Patients

Consecutive patients who had APS ICD-9 code and were admitted to the Department of Rheumatology and Immunology, Peking University People’s Hospital (PKUPH), were enrolled retrospectively in this study. The inclusion criteria were: 1) Patients fulfilled the 2006 Sydney criteria (2) (seropositive APS, SPAPS) or fulfilled the Sydney clinical criteria but were persistently negative for aCL, aβ2GPI, and LA at least on two separate occasions (seronegative APS, SNAPS) (8). At least one obstetric or one major non-obstetric or two minor non-obstetric “non-criteria” manifestations were also required for the classification of SNAPS (19). The obstetric, major, and minor non-obstetric “non-criteria” manifestations were shown in **Supplementary Tables 1**; 2) The serum of these patients were collected simultaneously and stored in -80°C freezer. The exclusion criteria were: 1) Patients with hereditary and other acquired thrombophilia disorders; 2) Incomplete medical records. At least two expert rheumatologists confirmed the diagnosis for patients.

The inclusion criteria of other autoimmune diseases were: 1) patients without thrombosis and pregnancy morbidity; 2) patients without APS. The patients with other autoimmune diseases were diagnosed according to current classification. The medical records were reviewed to obtain patients’ demographic and clinical information. Demographic data, clinical data, co-morbidity and laboratory data were collected.

This study was approved by the ethics committees of Peking University People’s Hospital (2019PHB253) and fulfilled the Declaration of Helsinki guidelines for the inclusion of humans in research.

### Detection of criteria aPLs

IgG, IgM and IgA isotypes of aCL and aβ2GPI were detected using quantitative IMTEC ELISA kits (HUMAN Diagnostics, Inc, Wiesbaden, GER). According to the manufacturer, the cutoff values of positive aCL and aβ2 GPI were 45U/ml and 5U/ml, which were consistent with the ROC curve calculated by HC.

The lupus anticoagulant test was conducted as previously described (20). The simplified Dilute Russell’s Viper Venom Test (dRVVT) was performed using the Stago STA Compact Hemostasis system. It used diluted activated partial thromboplastin time as screening tests by ISTH recommendations (21).

### Detection of non-criteria aPLs

Antibodies against phosphatidylserine/prothrombin (aPS/PT) IgG/IgA/IgM were measured using quantitative ELISA kits (HUMAN Diagnostics, Inc, Wiesbaden, GER). The cut-off value of aPS/PT was 30U/ml, according to the manufacturer’s instructions.

Anti-Annexin V antibodies (aAnnexinV) were detected using indirect solid-phase ELISA (HUMAN Diagnostics, Inc, Wiesbaden, GER) for the quantitative measurement of IgG, IgA, and IgM class autoantibodies against annexin V in human serum. Results above 25U/ml were considered positive.

Anti-phosphatidylethanolamine antibodies (aPE) IgG/IgA/IgM were measured using IMTEC ELISA kits with β2 GPI as a cofactor. According to the manufacturer’s instructions, the cutoff value was ≥ 15U/ml.

Anti-phosphatidylserine (aPS) IgM and IgG were measured using quantitative IMTEC ELISA kits (HUMAN Diagnostics, Inc, Wiesbaden, GER) against phosphatidylserine/β2GPI. Results above 15U/ml were considered positive for both IgM and IgG.

Antigens of APhL were a mixture of negatively charged phospholipids. The APhL IgG and APhL IgM were measured using the APhL ELISA assay (Louisville APL Diagnostic, Inc, Louisville, KY, USA), and cutoff values of 15 GPL/MPL units were used as recommended by the manufacturer.

### Statistical analysis

Variables with a normal distribution were presented as means with standard deviations or absolute numbers with percentages of the total. The data were presented as medians and interquartile ranges (IQRs) for variables with skewed distribution. The sensitivity, specificity, accuracy, Youden index, positive predictive value (PPV), negative predictive value (NPV), positive likelihood ratio (PLR), and negative likelihood ratio (NLR) were calculated for the diagnosis of APS. Logistic regression models were applied to assess the diagnostic values of different aPL combinations for APS. The receiver operating characteristic (ROC) curves were generated for single or combined aPLs, respectively. The area under the curve (AUC) were calculated to evaluate the diagnostic performance of the single or combined aPLs. Comparisons between non-criteria aPLs and clinical manifestations of APS were performed using the χ^2^ test. Fisher’s exact test was used if the expected number in a cell of a two-by-two table was less than five. Titers of non-criteria aPLs between groups were compared with the Mann-Whitney *U* test. The differences between groups were calculated by one-way ANOVA. Statistical significance was set at *p*-values less than 0.05. Statistical analysis was performed using SPSS v.15.0 (IBMCorp., Armonk, NY, USA) or R (version3.6.0).

## Results

A total of 347 patients were included in this study. Among these patients, 175 APS patients were categorized into APS groups, 122 patients with other autoimmune diseases without thrombosis or obstetrical morbidity, and 50 healthy controls (HC) served as the control group.

There were 115 SPAPS patients, with 94 (81.7%) females. The mean age was 42.4 years. Among these patients, 80 (69.6%) patients had a history of thrombosis, 42 (44.7%) female patients had a history of pregnancy morbidity and 7 (7.4%) female patients had a history of both thrombosis and pregnancy morbidity (**Table 1**).

**Table 1:** Demographic and clinical characteristics of the study population.

	SPAPS(N=115)	SNAPS(N=60)	OA(N=20)	RA(N=17)	SLE(N=42)	SS(N=26)	AS(N=17)	Healthy controls(N=50)
Mean age (years ± SD)	42.4 ± 15.3	38.2 ± 13.4	64.3 ± 12.6	60.3 ± 12.7	40.6 ± 15.1	58.2 ± 11.4	46.6 ± 16.1	42.4 ± 10.3
Sex (female), n (%)	94 (81.7)	55 (91.7)	17 (85.0)	16 (94.1)	40 (95.2)	26 (100.0)	7 (41.2)	33 (66.0)
Hypertension, n (%)	24 (20.9)	4 (6.7)	—	—	—	—	—	—
Diabetes mellitus, n (%)	11 (9.6)	2 (3.3)	—	—	—	—	—	—
Smoking status, n (%)	17 (14.8)	4 (6.7)	—	—	—	—	—	—
Newly diagnosed, n (%)	42 (36.5)	19 (31.7)	—	—	—	—	—	—
Thrombosis	80 (69.6)	25 (41.7)	—	—	—	—	—	—
Venous thrombosis, n (%)	38 (33.0)	17 (28.3)	—	—	—	—	—	—
DVT, n (%)	31 (27.0)	13 (21.7)	—	—	—	—	—	—
PE, n (%)	16 (13.9)	5 (8.3)	—	—	—	—	—	—
Arterial thrombosis, n (%)	61 (53.0)	15 (25.0)	—	—	—	—	—	—
Stroke, n (%)	30 (26.1)	6 (10.0)	—	—	—	—	—	—
CAD, n (%)	10 (8.7)	0	—	—	—	—	—	—
Both venous and arterial thrombosis, n (%)	19 (16.5)	7 (11.7)	—	—	—	—	—	—
Pregnancy morbidity, n (%)	42/94 (44.7)	43/55 (78.2)	—	—	—	—	—	—
Both thrombosis and pregnancy morbidity, n (%)	7/94 (7.4)	8/55 (14.5)	—	—	—	—	—	—
aCL (+), n (%)	29 (25.2)	0	0	0	0	1 (3.8)	0	0
aβ2GPI (+), n (%)	62 (53.9)	0	0	0	1 (2.4)	0	1 (5.9)	1 (2.0)
LA (+), n (%)	96 (83.5)	0	0	0	7 (16.7)	0	1 (5.9)	0
Double positive aPLs, n (%)	30 (26.1)	0	—	—	—	—	—	—
Triple positive aPLs, n (%)	21 (18.3)	0	—	—	—	—	—	—

SPAPS, seropositive antiphospholipid syndrome; SNAPS, seronegative antiphospholipid syndrome; OA, osteoarthritis; RA, rheumatoid arthritis; SLE, systemic lupus erythematosus; SS, Sjögren’s syndrome; AS, ankylosing spondylitis; DVT, deep venous thrombosis; PE, pulmonary embolism; AT, arterial thrombosis; CAD, coronary atherosclerotic heart disease; LA, lupus anticoagulant; aPLs, antiphospholipid antibodies.

Sixty patients were included in the SNAPS group. In this SNAPS group, 55 (91.7%) were female patients with mean age of 38.2 years. Thrombosis was present in 25 (41.7%) patients, and pregnancy morbidity was present in 43 (78.2%) female patients. In addition, 8 (14.5%) patients had a history of thrombosis and pregnancy morbidity (**Table 1**). The rate of non-criteria clinical manifestation of APS was presented in **Supplementary Table 2**.

The disease control (DC) group included 42 patients with systemic lupus erythematosus (SLE), 26 patients with Sjögren’s syndrome (SS), 17 patients with rheumatoid arthritis (RA), 17 patients with ankylosing spondylitis (AS), and 20 patients with osteoarthritis (OA). The baseline characteristics are presented in **Table 1**.

### Prevalence and diagnostic values of aPLs

The aCL, aβ2GPI, and LA were present in 29 (25.2%), 62 (53.9%), and 96 (83.5%) of the SPAPS patients, respectively. The prevalence of the seven non-criteria aPLs were shown in **Table 2**. For the non-criteria aPLs, the presence of aAnnexinV, aPE, aPS/PT, aPS IgG, aPS IgM, APhL IgG, and APhL IgM in the SPAPS patients were 67.0%, 40.9%, 60.0%, 53.9%, 20.0%, 55.7%, and 19.1%, respectively, and were significantly higher than in the control groups.

**Table 2:** Prevalence of non-criteria antibodies.

	SPAPS (N=115)	SNAPS (N=60)	Disease controls (N=122)	Healthy controls (N=50)	*p_1_^a^*	*p_2_^a^*
aAnnexinV, n (%)	77 (67.0)	26 (43.3)	68 (55.7)	8 (16.0)	0.000	0.904
aPE, n (%)	47 (40.9)	10 (16.7)	15 (12.3)	5 (10.0)	0.000	0.317
aPS/PT, n (%)	69 (60.0)	10 (16.7)	8 (6.6)	1 (2.0)	0.000	0.005
aPS IgG, n (%)	62 (53.9)	9 (15.0)	19 (15.6)	0	0.000	0.418
aPS IgM, n (%)	23 (20.0)	6 (10.0)	7 (5.7)	1 (2.0)	0.000	0.134
APhL IgG, n (%)	64 (55.7)	4 (6.7)	8 (6.6)	0	0.000	0.550
APhL IgM, n (%)	22 (19.1)	13 (21.7)	8 (6.6)	1 (2.0)	0.000	0.000

p_1_, p-values refer to SPAPS vs. HC and DC; p_2_, p-values refer to SNAPS vs. HC and DC; SPAPS, seropositive antiphospholipid syndrome; SNAPS, seronegative antiphospholipid syndrome; DC, disease control; HC, healthy control. ^a^Pearson Chi-square test.

The titers of these criteria and non-criteria aPLs among the different groups were illustrated in **Supplementary Figure 1**. No significant differences were observed in levels of aPS IgM and APhL IgM between SPAPS and SNAPS groups. Compared to the healthy controls, levels of all autoantibodies were significantly elevated in patients with SPAPS. Compared to the disease control groups, levels of aβ2GPI, aPS/PT, aPS IgG, and APhL IgG were increased dramatically in patients with SPAPS.

ACL exhibited the highest specificity of 99.42% but with a low sensitivity of 16.57%, followed by aβ2GPI (specificity of 98.26% and sensitivity of 34.29%). aAnnexinV exhibited the highest sensitivity of 58.86% but with the lowest specificity of 55.81%. LA displayed the highest Youden index of 0.5021 (specificity of 95.35% and sensitivity of 54.86%), followed by aPS/PT (Youden index of 0.3991)(**Table 3**). The diagnostic value for each APS subtypes were also analyzed. The Youden index of most criteria and non-criteria aPLs were higher in APS patients only with a history of thrombosis than in APS patients only with a history of pregnancy morbidity, and the same situation occurred between secondary and primary patients (**Supplementary Tables 3**–**5**).

**Table 3:** Diagnostic values of criteria and non-criteria antibodies.

	Sensitivity (%)	Specificity (%)	Accuracy (%)	Youden Index	PPV (%)	NPV (%)	OR (95%CI)	PLR	NLR
aCL	16.57	99.42	57.64	0.1599	96.67	53.94	33.97 (4.57, 252.40)	28.50	0.84
aβ2 GPI	34.29	98.26	65.99	0.3254	95.24	59.51	29.39 (9.00, 95.98)	19.66	0.67
LA	54.86	95.35	74.93	0.5021	92.31	67.49	24.91 (11.54, 53.78)	11.79	0.47
APhL IgG	38.86	95.35	66.86	0.3421	89.47	60.52	13.03 (6.02, 28.19)	8.35	0.64
aPS IgM	16.57	95.35	55.62	0.1192	78.38	52.90	4.07 (1.80, 9.19)	3.56	0.88
aPS/PT	45.14	94.77	69.74	0.3991	89.77	62.93	14.90 (7.15, 31.06)	8.63	0.58
APhL IgM	20.00	94.77	57.06	0.1477	79.55	53.79	4.53 (2.10, 9.75)	3.82	0.84
aPS IgG	41.04	88.95	64.93	0.2999	78.89	60.00	5.61 (3.19, 9.86)	3.72	0.66
aPE	32.57	88.37	60.23	0.2094	74.03	56.30	3.67 (2.09, 6.45)	2.80	0.76
aAnnexinV	58.86	55.81	57.35	0.1467	57.54	57.14	1.81 (1.18, 2.77)	1.33	0.74

PPV, positive predictive value; NPV, negative predictive value; OR, odds ratio; PLR, positive likelihood ratio; NLR, negative likelihood ratio; CI, confidence interval.

**Figure 1:**
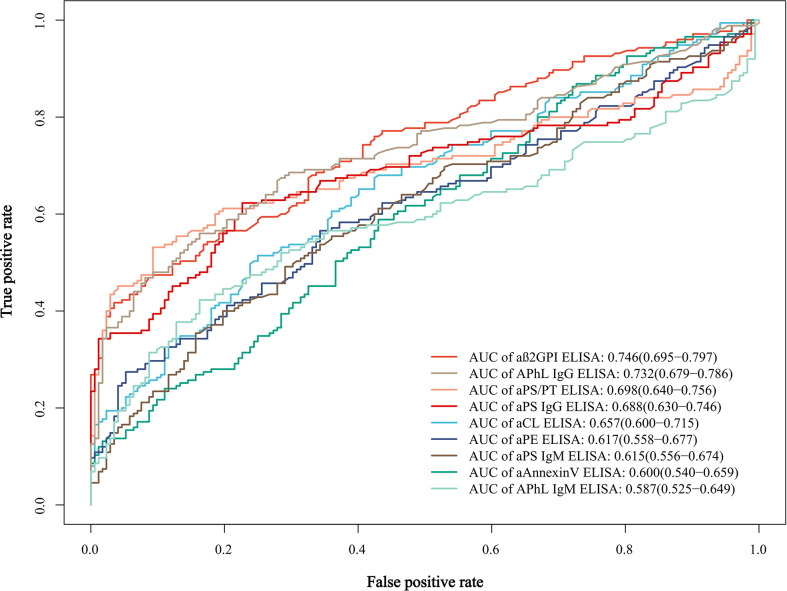
Receiver operating characteristic (ROC) curves and area under the curve (AUC) of the single aPLs. AUC values of each curve are shown with 95% confidence interval (CI) and are listed in a descending order.

To further evaluate the predictive value of these criteria and non-criteria aPLs for APS, the receiver operating characteristic (ROC) curves were plotted. Among these aPLs, aβ2GPI showed the most significant area under the curve (AUC = 0.746), followed by APhL IgG (AUC = 0.732) (**Figure 1** and **Supplementary Table 6**).

### Association between aPLs and clinical manifestations

Compared to the APS patients only with a history of pregnancy morbidity, the positivity and levels of aβ2GPI, aPS/PT, aPS IgG and APhL IgG were significantly increased in APS patients only with a history of thrombosis (**Table 4** and **Supplementary Figure 2**). Furthermore, the prevalence of LA in APS patients with a history of thrombosis only was significantly higher than in APS patients with a history of pregnancy morbidity only (**Table 4**).

**Table 4:** Prevalence of different antibodies among APS patients with a history of thrombosis only, pregnancy morbidity only, or both.

	Thrombosis only (N=90)	Pregnancy morbidity only (N=70)	Both (N=15)	*p_1_^a^*	*p_2_*	*p_3_*
aCL, n (%)	19 (21.1)	9 (12.9)	1 (6.7)	0.173	0.683^b^	0.293^b^
aβ2 GPI, n (%)	45 (50.0)	14 (20.0)	3 (20.0)	0.000*	1.000^b^	0.048^b,^*
LA, n (%)	59 (65.6)	31 (44.3)	6 (40.0)	0.007*	0.761^a^	0.059^a^
aAnnexinV, n (%)	52 (57.8)	41 (58.6)	10 (66.7)	0.920	0.561^a^	0.517^a^
aPE, n (%)	36 (40.0)	18 (25.7)	3 (20.0)	0.058	0.753^b^	0.161^b^
aPS/PT, n (%)	51 (56.7)	22 (31.4)	6 (40.0)	0.001*	0.522^a^	0.230^a^
aPS IgG, n (%)	45 (50.0)	22 (31.4)	4 (26.7)	0.015*	1.000^b^	0.156^b^
aPS IgM, n (%)	15 (16.7)	12 (17.1)	2 (13.3)	0.936	1.000^b^	1.000^b^
APhL IgG, n (%)	45 (50.0)	19 (27.1)	4 (26.7)	0.003*	1.000^b^	0.161^b^
APhL IgM, n (%)	16 (17.8)	18 (25.7)	1 (6.7)	0.223	0.172^b^	0.456^b^

p_1_, p-values refer to Thrombosis vs. Pregnancy morbidity; p_2_, p-values refer to Both vs. Pregnancy morbidity; p_3_, p-values refer to Thrombosis vs. Both. ^a^Pearson Chi-square test; ^b^Fisher’s exact test. *p < 0.05.

Presence of thrombosis was significantly associated with aPS/PT [odds ratio (OR) 2.48, 95% confidence interval (CI) 1.32-4.69, *p* = 0.004], aPS IgG (OR 1.90, 95%CI 1.01-3.60, *p* = 0.046), and APhL IgG (OR 2.26, 95%CI 1.18-4.34, *p* = 0.013). Arterial thrombosis was significantly associated with aPS/PT (OR 2.28, 95%CI 1.24-4.20, *p* = 0.008), aPS IgG (OR 2.58, 95%CI 1.38-4.82, *p* = 0.003), and APhL IgG (OR 2.54, 95%CI 1.36-4.75, *p* = 0.003). Additionally, stroke was significantly associated with aPS/PT (OR 2.23, 95%CI 1.05-4.73, *p* = 0.034) and aPS IgG (OR 2.44, 95%CI 1.13-5.24, *p* = 0.020).

### Added values of different aPLs in the diagnosis of APS and SNAPS

As shown in **Table 2**, the prevalence of aAnnexinV, aPE, aPS/PT, aPS IgG, aPS IgM, APhL IgG, and APhL IgM in SNAPS patients were 43.3%, 16.7%, 16.7%, 15.0%, 10.0%, 6.7%, and 21.7%, respectively. By adding the “non-criteria” aPLs, the aPL positive rate was increased from 65.7% (criteria aPLs only) to 87.4% in APS patients (**Supplementary Figure 3**).

**Figure 2:**
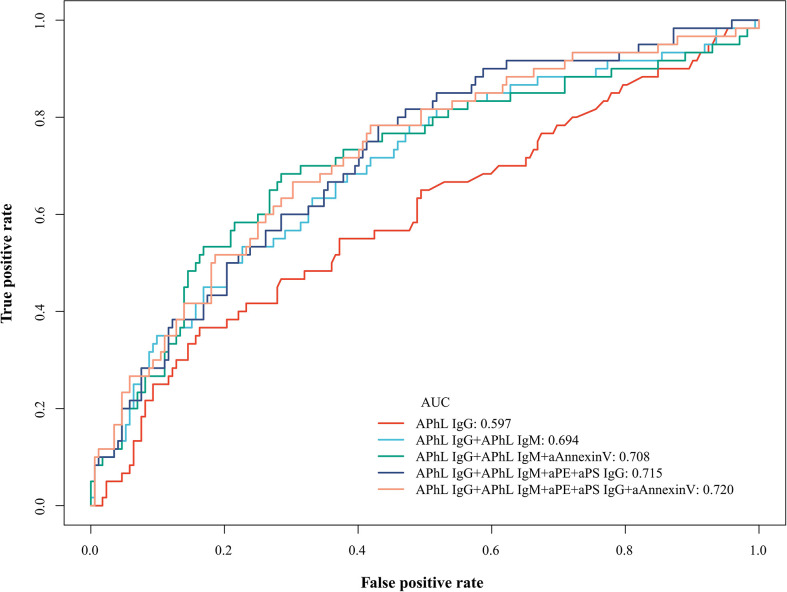
Receiver operating characteristic (ROC) curves and area under the curve (AUC) of single or combined non-criteria antibodies among SNAPS patients and controls. In seven single antibodies and combinations of two to five antibodies, the ROC curves with the highest AUC values are shown. Among SNAPS patients, the APhL IgG showed the highest AUC value in single antibodies. The APhL IgG/IgM showed the highest AUC among two antibody combinations, and the same is true of the other three combinations.

Seven single antibodies or two to five antibody combinations were analyzed among SNAPS patients, respectively. The ROC curves were applied to evaluate the predictive value, and the ones with the highest AUC values were shown in **Figure 2** and **Supplementary Table 7**. The APhL IgG showed the highest AUC of 0.597 among single non-criteria aPLs in SNAPS patients. The APhL IgG/IgM showed the highest AUC of 0.694 among two antibody combinations. The APhL IgG/IgM plus aAnnexinV showed the highest AUC of 0.708 among three antibody combinations. The APhL IgG/IgM, aPS IgG plus aPE showed the highest AUC of 0.715 among four antibody combinations. The APhL IgG/IgM, aPS IgG, aPE plus aAnnexinV showed the highest AUC of 0.720 among five antibody combinations.

## Discussion

This study assessed the clinical significance of non-criteria aPLs in APS. Among these non-criteria aPLs, aAnnexin V showed the highest sensitivity, while APhL and aPS IgM showed the highest specificity. APhL, aPS/PT, and aPS IgG may be potential biomarkers to predict thrombotic risk in APS.

It has been reported that aPS/PT was a useful diagnostic marker for thrombosis in APS (22), especially for arterial thrombosis. Among 323 patients with or without APS who tested for aPLs, aPS/PT could additionally identify 2% of obstetric patients and 3% of thrombotic patients (17). The positive rate of aPS/PT was 16.7% in SNAPS in our study, it is also a valuable marker for SNAPS (13), and could additionally identify 9% of obstetric patients and 5% of thrombotic patients among patients with APS. Therefore, aPS/PT can be used as a diagnostic marker in APS and may also indicate thrombosis.

Annexin V is a potent anticoagulant protein by its ability to bind phospholipids, form crystals and block the availability of phospholipids to phospholipid-dependent coagulation enzymes (23, 24). According to Pooled Data from three studies, Annexin V resistance was present in more than half of patients with APS (9). And the aPL-mediated reduction of Annexin V has been observed on placental trophoblasts (25) and endothelial cells (26–28). The presence of aAnnexinV may impair the anticoagulant shield and lead to thrombosis and pregnancy morbidity. Although aAnnexinV might be involved in the pathogenesis of APS, it appeared that conflicting conclusions were observed between aAnnexinV and clinical features (16, 29 , 23, 30–33). In our study, the clinical significance of aAnnexinV is not as predictive as other non-criteria aPLs. Still, combinations of aAnnexinV and other non-criteria antibodies may better recognize patients with seronegative APS.

This study evaluated the clinical significance of antibodies against phospholipid antigens, including aPE, aPS, and APhL. The antibodies against phospholipid antigens include β2-GPI-dependent (β2-GPI-dependent) and β2-GPI-independent forms. The aPLs in the serum of patients with infectious diseases are β2-GPI-independent, which are unrelated to thrombosis (34). The β2-GPI-dependent aPLs were more specific to APS. Thus, β2-GPI was a cofactor for aPE (12) and aPS (35, 36). There were no associations between aPE and clinical manifestations in our and other APS cohorts (37, 38). Therefore, aPE may not serve as a marker for thrombosis or pregnancy morbidity in APS. The diagnostic value of aPS revealed high sensitivity and specificity (35), and it was associated with thrombosis. Therefore, aPS may serve as a diagnostic indicator for APS.

APhL reduced the false positives associated with the aCL test and improved the specificity in diagnosing APS (39). The prevalence of APhL was 11.5% and the specificity was 92.8 to 97.6% (40). APhL was associated with arterial thrombosis and pregnancy-related morbidity (40). We confirmed the association between APhL IgG and arterial thrombosis. APhL IgG is also a promising biomarker for SNAPS.

The combined autoantibodies tests might help to increase the sensitivity in the diagnosis of APS, but decrease the specificity (16). In our study, the sensitivity increased to 87.4% after adding all these 7 non-criteria aPLs. The presence of any 7 or more aPLs was linked with arterial thrombosis with an odds ratio (OR) of 4.1 (41). A longitudinal study conducted for 15 years showed that the risk of thrombosis progressively increased with the number of positive aPLs (42). The risk of thrombosis increased to thirtyfold higher after adding 4 positive antibody tests (42). Patients with catastrophic APS, a severe APS, had the highest number of non-criteria aPLs (43). Therefore, aPL profiling is more important than single aPL tests in APS diagnosis and risk stratification.

In clinical practice, it is unlikely to test all non-criteria aPLs but reasonably only includes highly specific non-criteria aPLs. It will improve the diagnostic accuracy for APS. In this study, we demonstrated that the aPS/PT, aPS and APhL could be three promising markers for diagnosing APS.

This study has some limitations. First, we didn’t include patients with recurrent thrombosis or pregnancy morbidity without APS as disease controls. Further studies are needed to evaluate the clinical significance of these aPLs. Second, the sample size of SNAPS patients was relatively small. This sample requires verification with a larger population for the diagnostic utility of non-criteria aPLs in SNAPS. Third, this is a single-center study. In the future, it will be worthwhile to initiate a multicenter investigation with a larger sample size to determine how consistently the non-criteria aPLs improve the diagnostic accuracy in APS.

## Conclusions

Several non-criteria aPLs were significantly increased in patients with APS. These non-criteria aPLs could improve the diagnostic value for APS. Detecting aPS/PT, aPS, and APhL may serve as reliable markers to predict the risk of SNAPS and thrombosis.

## Data availability statement

The raw data supporting the conclusions of this article will be made available by the authors, without undue reservation.

## Ethics statement

The studies involving human participants were reviewed and approved by the ethic committees of Peking University People’s Hospital (2019PHB253). The patients/participants provided their written informed consent to participate in this study.

## Author contributions

All authors were involved in drafting and revising the manuscript, and all authors approved the final version to be submitted for publication. XL and LZ collected and analyzed data. LZ, HL, QC, and FS performed the ELISA assays. XL, LZ, HL, ZY, YJ, CL, and JG performed the statistical analysis and wrote the manuscript.

## Funding

This work was supported in part by the China International Medical Foundation (No. Z-2018-40-2101), the National Natural Science Foundation of China (No. 81871281 and 31870913) and the Nantong Science and Technology Project (No. MSZ20004).

## Acknowledgments

We thank the patients and healthy volunteers for their cooperation.

## Conflict of interest

The authors declare that the research was conducted in the absence of any commercial or financial relationships that could be construed as a potential conflict of interest.

## Publisher’s note

All claims expressed in this article are solely those of the authors and do not necessarily represent those of their affiliated organizations, or those of the publisher, the editors and the reviewers. Any product that may be evaluated in this article, or claim that may be made by its manufacturer, is not guaranteed or endorsed by the publisher.

## Supplementary material

Supplementary Figure 1 Level of criteria and non-criteria antibodies in patients with antiphospholipid syndrome (APS) and in control subjects as determined by ELISA. Dot plot of the nine antibody titers among different diagnostic groups, with the lines showing the quartile values. *P-*values by the Mann-Whitney *U* test. SPAPS, seropositive antiphospholipid syndrome; SNAPS, seronegative antiphospholipid syndrome; OA, osteoarthritis; RA, rheumatoid arthritis; SLE, systemic lupus erythematosus; SS, Sjögren’s syndrome; AS, ankylosing spondylitis; HC, healthy controls. **p* < 0.05, ***p* < 0.01, ****p* < 0.001; NS, not significant.

Supplementary Figure 2 Level of different antibodies in APS patients with a history of thrombosis only, pregnancy morbidity only, or both. Dot plot of the nine antibody titers among groups with different clinical manifestations, with the lines showing the quartile values. The *p*-values from the Mann-Whitney *U* test. **p* < 0.05, ***p* < 0.01, ****p* < 0.001; NS, not significant.

Supplementary Figure 3 Diagnostic values among criteria and non-criteria antibodies. By adding the “non-criteria” aPLs, the aPL positive rate was increased from 65.7% (criteria aPLs only) to 87.4% in APS patients.
