# Supplementary material for: Non-criteria antiphospholipid antibodies in antiphospholipid syndrome: Diagnostic value added
Source: Front Immunol. 2022 Oct 26;13:972012. doi: 10.3389/fimmu.2022.972012 (PMC9643638; doi:10.3389/fimmu.2022.972012)
Supplement: Supplementary file 1 [file Table_1.docx]

**Supplementary Table 1** Included “non-criteria” manifestations of APS^[1]^

| **Non-obstetric manifestations** |  | **Obstetric** |
| --- | --- | --- |
| **Major** |  | Infertility |
| Acute ischemic encephalopathy | Adrenal hemorrhage | Late IUGR (after 34 weeks) |
| APS nephropathy | Cardiac microvascular disease | Late pre-eclampsia (after 34 weeks) |
| Chorea | Evans syndrome | Placental abruption |
| *Livedo reticularis/racemosa* | Livedoid vasculopathy | Placental hematoma |
| Longitudinal myelitis | Pulmonary hemorrhage | Preterm birth (>34 to <37 weeks) |
| Superfcial vein thrombosis | Thrombocytopenia | Puerperal pre-eclampsia |
| Valvular heart disease |  | Two or more unexplained in vitro fertilization failures |
| **Minor** |  | Two unexplained spontaneous abortions <10 weeks |
| *Amaurosis fugax* | Brain MRI white matter lesions |  |
| Cognitive dysfunction | Coombs’ test positivity |  |
| Hemolytic anemia | Ischemic necrosis of bone |  |
| Migraine | Pseudo-multiple sclerosis |  |
| Pulmonary hypertension | Raynaud’s phenomenon |  |
| Seizures | Sensorineural hearing loss |  |
| Splinter hemorrhages |  |  |

APS, Antiphospholipid syndrome; IUGR, Intrauterine growth restriction.

References:

[1] da Rosa GP, Sousa-Pinto B, Ferreira E, et al. The presence of non-criteria manifestations negatively affects the prognosis of seronegative antiphospholipid syndrome patients: a multicenter study. Arthritis Res Ther. 2022. 24(1): 9.

**Supplementary Table 2** The rate of every non-criteria clinical manifestation of APS

| **Non-criteria manifestations** | **APS, n (%)** |
| --- | --- |
| **Non-obstetric manifestations** |  |
| **Major** |  |
| Acute ischemic encephalopathy | 1 (0.6) |
| APS nephropathy | 1 (0.6) |
| Chorea | - |
| *Livedo reticularis/racemosa* | 2 (1.1) |
| Longitudinal myelitis | - |
| Superfcial vein thrombosis | 5 (2.9) |
| Valvular heart disease | 3 (1.7) |
| Adrenal hemorrhage | 2 (1.1) |
| Cardiac microvascular disease | - |
| Evans syndrome | 9 (5.1) |
| Livedoid vasculopathy | - |
| Pulmonary hemorrhage | 3 (1.7) |
| Thrombocytopenia | 82 (46.9) |
| **Minor** |  |
| *Amaurosis fugax* | 1 (0.6) |
| Cognitive dysfunction | 2 (1.1) |
| Hemolytic anemia | 14 (8.0) |
| Migraine | - |
| Pulmonary hypertension | 3 (1.7) |
| Seizures | 9 (5.1) |
| Splinter hemorrhages | - |
| Brain MRI white matter lesions | 13 (7.4) |
| Coombs’ test positivity | 23 (13.1) |
| Ischemic necrosis of bone | 2 (1.1) |
| Pseudo-multiple sclerosis | - |
| Raynaud’s phenomenon | 12 (6.9) |
| Sensorineural hearing loss | 1 (0.6) |
| **Obstetric** |  |
| Infertility | 4 (2.3) |
| Late IUGR (after 34 weeks) | 2 (1.1) |
| Late pre-eclampsia (after 34 weeks) | - |
| Placental abruption | - |
| Placental hematoma | - |
| Preterm birth (>34 to <37 weeks) | 3 (1.7) |
| Puerperal pre-eclampsia | - |
| Two or more unexplained in vitro fertilization failures | 3 (1.7) |
| Two unexplained spontaneous abortions <10 weeks | 7 (4.0) |

APS, Antiphospholipid syndrome**.**

**Supplementary Table 3** Diagnostic values of criteria and non-criteria antibodies among APS patients with a history of thrombosis only.

|  | Sensitivity  (%) | Specificity  (%) | Accuracy  (%) | Youden Index | PPV  (%) | NPV  (%) | OR(95%CI) | PLR | NLR |
| --- | --- | --- | --- | --- | --- | --- | --- | --- | --- |
| aCL | 21.11 | 99.42 | 72.52 | 0.2053 | 95.00 | 70.66 | 45.76 (6.01, 348.37) | 36.31 | 0.79 |
| aβ2 GPI | 50.00 | 98.84 | 82.06 | 0.4884 | 95.74 | 50.59 | 85.00 (19.86, 363.81) | 43.00 | 0.51 |
| LA | 65.56 | 95.35 | 85.11 | 0.6090 | 88.06 | 84.10 | 39.02 (16.97, 89.68) | 14.09 | 0.36 |
| APhL IgG | 50.00 | 95.32 | 79.69 | 0.4532 | 84.91 | 78.37 | 20.38 (8.96, 46.32) | 10.69 | 0.52 |
| aPS IgM | 16.67 | 95.35 | 68.32 | 0.1202 | 65.22 | 68.62 | 4.10 (1.67, 10.09) | 3.58 | 0.87 |
| aPS/PT | 56.67 | 94.77 | 81.68 | 0.5143 | 85.00 | 80.69 | 23.68 (10.75, 52.19) | 10.83 | 0.46 |
| APhL IgM | 17.78 | 94.74 | 68.20 | 0.1251 | 64.00 | 68.64 | 3.89 (1.64, 9.21) | 3.38 | 0.87 |
| aPS IgG | 50.56 | 88.95 | 75.86 | 0.3952 | 70.31 | 77.66 | 8.24 (4.38, 15.50) | 4.58 | 0.56 |

| aPE | 40.00 | 88.37 | 71.76 | 0.2837 | 64.29 | 73.79 | 5.07 (2.70, 9.50) | 3.44 | 0.68 |
| --- | --- | --- | --- | --- | --- | --- | --- | --- | --- |

| aAnnexinV | 57.78 | 55.81 | 56.49 | 0.1359 | 40.62 | 71.64 | 1.73 (1.03, 2.89) | 1.31 | 0.76 |
| --- | --- | --- | --- | --- | --- | --- | --- | --- | --- |

PPV, positive predictive value; NPV, negative predictive value; OR, odds ratio; PLR, positive likelihood ratio; NLR, negative likelihood ratio; CI, confidence interval; aCL, anticardiolipin antibodies; aβ2 GPI, anti-β2-glycoprotein I antibodies; LA, lupus anticoagulant; APhL, antibodies directed against a mixture of phospholipids; aPS, anti-phosphatidylserine antibodies; aPS/PT, anti-phosphatidylserine/prothrombin antibodies; aPE, anti-phosphatidylethanolamine antibodies; aAnnexinV, anti-Annexin V antibodies.

**Supplementary Table 4** Diagnostic values of criteria and non-criteria antibodies among APS patients with a history of pregnancy morbidity only.

|  | Sensitivity  (%) | Specificity  (%) | Accuracy  (%) | Youden Index | PPV  (%) | NPV  (%) | OR(95%CI) | PLR | NLR |
| --- | --- | --- | --- | --- | --- | --- | --- | --- | --- |
| aCL | 12.86 | 99.42 | 74.38 | 0.1228 | 90.00 | 73.71 | 25.23 (3.13, 203.28) | 22.11 | 0.88 |
| aβ2 GPI | 20.00 | 98.84 | 76.03 | 0.1884 | 87.50 | 75.22 | 21.25 (4.68, 96.39) | 17.20 | 0.81 |
| LA | 44.29 | 95.35 | 80.58 | 0.3963 | 79.49 | 80.79 | 16.29 (6.95, 38.20) | 9.52 | 0.58 |
| APhL IgG | 27.14 | 95.32 | 75.52 | 0.2246 | 70.37 | 76.17 | 7.59 (3.14, 18.37) | 5.80 | 76.43 |
| aPS IgM | 17.14 | 95.35 | 72.73 | 0.1249 | 60.00 | 73.87 | 4.24 (1.65, 10.89) | 3.69 | 0.87 |
| aPS/PT | 31.43 | 94.77 | 76.45 | 0.2620 | 70.97 | 77.25 | 8.30 (3.58, 19.22) | 6.01 | 0.72 |
| APhL IgM | 25.71 | 94.74 | 74.69 | 0.2045 | 66.67 | 75.70 | 6.23 (2.64, 14.71) | 4.89 | 0.78 |
| aPS IgG | 31.43 | 88.95 | 72.31 | 0.2038 | 53.66 | 76.12 | 3.69 (1.84, 7.39) | 2.85 | 0.77 |

| aPE | 25.71 | 88.37 | 70.25 | 0.1409 | 47.37 | 74.51 | 2.63 (1.29, 5.35) | 2.21 | 0.84 |
| --- | --- | --- | --- | --- | --- | --- | --- | --- | --- |

| aAnnexinV | 58.57 | 55.81 | 56.61 | 0.1439 | 35.04 | 76.80 | 1.79 (1.02, 3.14) | 1.33 | 0.74 |
| --- | --- | --- | --- | --- | --- | --- | --- | --- | --- |

PPV, positive predictive value; NPV, negative predictive value; OR, odds ratio; PLR, positive likelihood ratio; NLR, negative likelihood ratio; CI, confidence interval; aCL, anticardiolipin antibodies; aβ2 GPI, anti-β2-glycoprotein I antibodies; LA, lupus anticoagulant; APhL, antibodies directed against a mixture of phospholipids; aPS, anti-phosphatidylserine antibodies; aPS/PT, anti-phosphatidylserine/prothrombin antibodies; aPE, anti-phosphatidylethanolamine antibodies; aAnnexinV, anti-Annexin V antibodies.

**Supplementary Table 5** Diagnostic values of criteria and non-criteria antibodies among secondary APS patients.

|  | Sensitivity  (%) | Specificity  (%) | Accuracy  (%) | Youden Index | PPV  (%) | NPV  (%) | OR(95%CI) | PLR | NLR |
| --- | --- | --- | --- | --- | --- | --- | --- | --- | --- |
| aCL | 18.68 | 99.42 | 71.48 | 0.1810 | 94.44 | 69.80 | 39.28 (5.13, 300.65) | 32.13 | 0.82 |
| aβ2 GPI | 40.66 | 98.84 | 78.71 | 0.3950 | 94.87 | 75.89 | 58.24 (13.59, 249.64) | 34.97 | 0.60 |
| LA | 63.74 | 95.35 | 84.41 | 0.5909 | 87.88 | 83.25 | 36.03 (15.74, 82.50) | 13.70 | 0.38 |
| APhL IgG | 41.76 | 95.32 | 76.72 | 0.3708 | 82.61 | 75.46 | 14.61 (6.41, 33.27) | 8.93 | 0.61 |
| aPS IgM | 18.68 | 95.35 | 68.82 | 0.1403 | 68.00 | 68.91 | 4.71 (1.95, 11.40) | 4.02 | 0.85 |
| aPS/PT | 53.85 | 94.77 | 80.61 | 0.4861 | 84.48 | 79.51 | 21.13 (9.61, 46.44) | 10.29 | 0.49 |
| APhL IgM | 19.78 | 94.74 | 68.70 | 0.1452 | 66.67 | 68.94 | 4.44 (1.90, 10.35) | 3.76 | 0.85 |
| aPS IgG | 44.44 | 88.95 | 73.66 | 0.3340 | 67.80 | 75.37 | 6.44 (3.42, 12.13) | 4.02 | 0.62 |

| aPE | 36.26 | 88.37 | 70.34 | 0.2464 | 62.26 | 72.38 | 4.32 (2.30, 8.14) | 3.12 | 0.72 |
| --- | --- | --- | --- | --- | --- | --- | --- | --- | --- |

| aAnnexinV | 58.24 | 55.81 | 56.65 | 0.1406 | 41.09 | 71.64 | 1.76 (1.05, 2.95) | 1.32 | 0.75 |
| --- | --- | --- | --- | --- | --- | --- | --- | --- | --- |

PPV, positive predictive value; NPV, negative predictive value; OR, odds ratio; PLR, positive likelihood ratio; NLR, negative likelihood ratio; CI, confidence interval; aCL, anticardiolipin antibodies; aβ2 GPI, anti-β2-glycoprotein I antibodies; LA, lupus anticoagulant; APhL, antibodies directed against a mixture of phospholipids; aPS, anti-phosphatidylserine antibodies; aPS/PT, anti-phosphatidylserine/prothrombin antibodies; aPE, anti-phosphatidylethanolamine antibodies; aAnnexinV, anti-Annexin V antibodies.

**Supplementary Table 6** Area under the curve (AUC) of the single aPLs and the 95% confidence interval (CI).

|  | AUC | 95% CI |
| --- | --- | --- |
| aβ2 GPI | 0.746 | 0.695-0.797 |
| APhL IgG | 0.732 | 0.679-0.786 |
| aPS/PT | 0.698 | 0.640-0.756 |
| aPS IgG | 0.688 | 0.630-0.746 |
| aCL | 0.657 | 0.600-0.715 |
| aPE | 0.617 | 0.558-0.677 |
| aPS IgM | 0.615 | 0.556-0.674 |
| aAnnexinV | 0.600 | 0.540-0.659 |
| APhL IgM | 0.587 | 0.525-0.649 |

AUC, area under the curve; CI, confidence interval; aβ2 GPI, anti-β2-glycoprotein I antibodies; APhL, antibodies directed against a mixture of phospholipids; aPS/PT, anti-phosphatidylserine/prothrombin antibodies; aPS, anti-phosphatidylserine antibodies; aCL, anticardiolipin antibodies; aPE, anti-phosphatidylethanolamine antibodies; aAnnexinV, anti-Annexin V antibodies.

**Supplementary Table 7** Area under the curve (AUC) of single or combined non-criteria antibodies among SNAPS patients and controls

| Individual antibodies or combinations | AUC |
| --- | --- |
| APhL IgG | 0.597 |
| APhL IgG+APhL IgM | 0.694 |
| APhL IgG+APhL IgM+aAnnexinV | 0.708 |
| APhL IgG+APhL IgM+aPE+aPS IgG | 0.715 |
| APhL IgG+APhL IgM+aPE+aPS IgG+aAnnexinV | 0.720 |

APhL, antibodies directed against a mixture of phospholipids; aAnnexinV, anti-Annexin V antibodies; aPS, anti-phosphatidylserine antibodies; aPE, anti-phosphatidylethanolamine antibodies.
