# Supplementary figures and images for: Non-criteria antiphospholipid antibodies in antiphospholipid syndrome: Diagnostic value added
Source: Front Immunol. 2022 Oct 26;13:972012. doi: 10.3389/fimmu.2022.972012 (PMC9643638; doi:10.3389/fimmu.2022.972012)

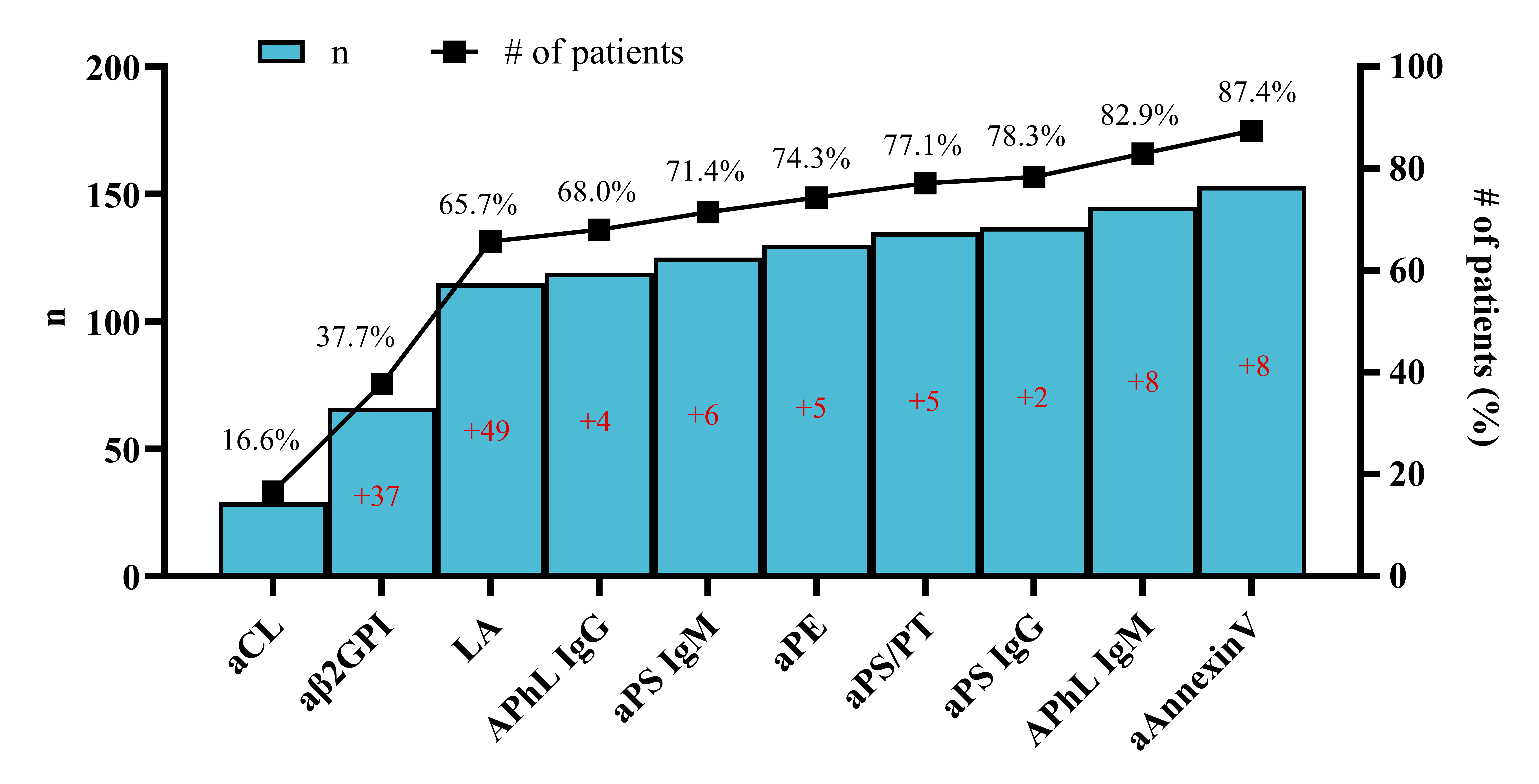

Supplement: Supplementary Figure 3 — Diagnostic values among criteria and non-criteria antibodies. By adding the “non-criteria” aPLs, the aPL positive rate was increased from 65.7% (criteria aPLs only) to 87.4% in APS patients. [file Image_3.tif]
